# Association between melatonin receptor gene polymorphisms and polycystic ovarian syndrome: a systematic review and meta-analysis

**DOI:** 10.1042/BSR20200824

**Published:** 2020-06-25

**Authors:** Shiqi Yi, Jiawei Xu, Hao Shi, Wenbo Li, Qian Li, Ying-pu Sun

**Affiliations:** 1 Center for Reproductive Medicine, The First Affiliated Hospital of Zhengzhou University, Zhengzhou, China; 2 Henan Key Laboratory of Reproduction and Genetics, The First Affiliated Hospital of Zhengzhou University, Zhengzhou, China; 3 Henan Provincial Obstetrical and Gynecological Diseases (Reproductive Medicine) Clinical Research Center, The First Affiliated Hospital of Zhengzhou University, Zhengzhou, China; 4 Henan Engineering Laboratory of Preimplantation Genetic Diagnosis and Screening, The First Affiliated Hospital of Zhengzhou University, Zhengzhou, China

**Keywords:** melatonin receptor, meta analysis, polycystic ovarian syndrome, single nucleotide polymorphisms

## Abstract

**Background:** Polycystic ovarian syndrome (PCOS) is a kind of common gynecological endocrine disorder. And the mutations of melatonin receptor (MTNR) genes are related to the occurrence of PCOS. But previous researches have shown opposite results. So, the object of our systematic review and meta-analysis is to investigate the relationship between MTNR 1A/B polymorphisms and PCOS.

**Methods:** PubMed, Embase, Ovid, the Cochrane Library, Web of Science and three Chinese databases (VIP, CNKI and Wanfang) were used to retrieve eligible articles published between January 1980 and February 2020. And we used the odds ratio (OR) and its 95% confidence interval (CI) to investigate the strength of the association by six genetic models, allelic, codominant (homozygous and heterozygous), dominant, recessive and superdominant models. Review Manager 5.3, IBM SPSS statistics 25 and Stata MP 16.0 software were used to do this meta-analysis.

**Results:** Our meta-analysis involved 2553 PCOS patients and 3152 controls, for two single nucleotide polymorphisms (rs10830963 C> G in MTNR1B and rs2119882 T> C in MTNR1A) and significant associations were found in some genetic models of these single nucleotide polymorphisms (SNPs). For rs10830963, strongly significant was found in the heterozygote model (GC vs. CC, *P*=0.02). Additionally, a slight trend was detected in the allelic (G vs. C), homozygote (GG vs. CC) and dominant (GG+GC vs. CC) model of rs10830963 (*P*=0.05). And after further sensitivity analysis, a study with high heterogeneity was removed. In the allelic (*P*=0.000), homozygote (*P*=0.001), dominant (*P*=0.000) and recessive (GG vs. GC+CC, *P*=0.001) model, strong associations between rs10830963 and PCOS were found. Moreover, for rs2119882, five genetic models, allelic (C vs. T, *P*=0.000), codominant (the homozygote (CC vs. TT, *P*=0.000) and heterozygote model (CT vs. TT, *P*=0.02), dominant (CC + CT vs. TT, *P*=0.03) and recessive model (CC vs. CT + TT, *P*=0.000) showed significant statistical associations with PCOS.

**Conclusion:** MTNR1B rs10830963 and MTNR1B rs2119882 polymorphisms are associated with PCOS risk. However, the above conclusions still require being confirmed by much larger multi-ethnic studies.

## Background

Melatonin secreted by the pineal gland during the dark phase of the sleep/wake cycle, is a kind of hormone which periodically regulates several physiological functions [1], including glucose homeostasis [2] and insulin secretion [3]. When the melatonin secretion or the corresponding Melatonin Receptor (MTNR) is abnormal, the metabolism in humans may be seriously damaged [4]. And MTNR exists in ovarian granulosa cells membranes [5,6], therefore, melatonin as a pleiotropic molecule has a direct effect on ovarian function [7,8]. Low melatonin levels in follicular fluid affect the quality and number of oocytes, and ultimately affect the outcome of *in vitro* fertilization (IVF) [9]. Moreover, melatonin treatment can also be used as a combination therapy to help control the blood glucose level [2], slow the progress or improve type 2 diabetes mellitus (T2DM) [10], increase pregnancy rates and improve endocrine levels eventually [11].

Additionally, multiple studies reveal that melatonin secretion is increased in polycystic ovarian syndrome (PCOS) patients [12–14]. PCOS is a kind of common metabolic and endocrine disorder in reproductive women [15], of which global prevalence is approximately 6–10% [16,17]. It has a variety of heterogeneous clinical manifestations, and approximately 20–30% of patients with PCOS suffer from various complications [18], like metabolic syndrome [19] and insulin resistance (IR) [20], which makes PCOS hard to be explained by a single factor. PCOS is a highly clinical as well as genetic heterogeneity syndrome [21]. For example, oocytes of PCOS patients also have abnormal gene transcription and expression, which will affect individual reproductive capacity [22]. As metabolic disorders have become much more prevalent recently [23], more genome-wide association and cohort studies were conducted and more variant genes and novel single nucleotide polymorphisms (SNPs) are found in large Chinese PCOS population [24,25], which have been confirmed later by multi-ethnic studies [26,27], including thyroid associated protein gene [28], Fat Mass and Obesity (*FTO*) gene [29,30], Follicle-stimulating Hormone Receptor (*FSHR*) gene [31,32], DENN/MADD domain containing 1A gene [33], Vitamin D Receptor (*VDR*) gene [34–36] and so on.

Additionally, the SNPs of MTNR 1A/B gene (MTNR1A/B) are found to have associations with many kinds of metabolic disorders. For example, MTNR1B rs10830963 polymorphism is not only associated with fasting blood glucose levels, but also with IR and T2DM risk [37]. And MTNR1A rs2119882 is also associated with gestational diabetes mellitus and IR [38]. However, whether MTNR polymorphisms relate to another metabolic disorder, PCOS, is still inconclusive because previous genetic research has shown conflicting conclusions [39]. So, the object of our meta-analysis is to ascertain the association between MTNR1A/B polymorphisms and PCOS.

## Methods

Our study was conducted with the requirements of Preferred Reporting Items for Systematic Reviews and Meta-Analyses (PRISMA) [40]. All data were available from currently published studies, so no informed consent and Ethics Committee approval were required.

### Study protocol

Five English databases (PubMed, Embase, Ovid, the Cochrane Library and Web of Science) and three Chinese databases (VIP, CNKI and Wanfang) were searched in our study. And we used three subject heading terms, ‘Polycystic ovarian syndrome’, ‘melatonin’ and ‘melatonin receptor’, as well as the synonyms of these terms confirmed by Medical Subject Headings (MeSH) referred to in the Supplementary Material S1 (Search strategies) [41], where other free words were registered. The latest search results were updated on 14 February 2020. And there were no language restrictions in these search strategies. Finally, all references were exported to EndNote X9 software for further research.

### Definitions and results

As the definition of PCOS used in the present study, all the eligible studies adopted the 2003 Rotterdam diagnostic inclusion criteria of PCOS [42]: (1) thin ovulation or anovulation. (2) Clinical manifestations of hyperandrogen or hyperandrogenemia. (3) Polycystic ovarian changes, that is, ≥12 follicles with a diameter of 2–9 millimeters on ovary, or ovarian volume > 10 ml. As long as two of the above criteria are met and other diseases are excluded, PCOS can be diagnosed. The results were the genotype distribution in MTNR gene and the odds ratio (OR) of PCOS.

### Eligibility criteria

Included references needed to meet the following criteria:

1. Assess the association between SNP in MTNR and PCOS risk,
2. Contain particular data, such as the frequency of MTNR genotypes.

Excluded studies should meet the following criteria:

1. Abstracts and reviews,
2. Repeated dissertation,
3. No research on genotype distribution,
4. Non-human research.

Among them, Newcastle–Ottawa Scale (NOS), available from URL (http://www.ohri.ca/programs/clinical_epidemiology/oxford.htm) [43], was performed to evaluate the quality of all included studies.

### Study selection

All studies retrieved from all the databases were assessed and reviewed by the first and second authors (Shiqi Yi and Hao Shi) based on eligibility criteria. The data extraction results were agreed upon discussion with another first author (Jiawei Xu). Study and descriptors such as author, publication time, country, ethnicity, study period, study type, NOS scores, the number of participants and methods of study were extracted and recorded from the included studies.

The third and fourth authors (Wenbo Li and Qian Li) independently assessed the quality of eligible studies using NOS which consisted of three parts, namely selection, comparability and exposure, and their corresponding eight scoring items. Except for comparability which could score up to two stars, each item could only score up to one star. Possible maximum number of stars in each study was 9. When the score of eligible study in NOS was ≥7, the present study was regarded as high-quality research [44]. When scoring divergences occurred, the sixth investigator (Ying-pu Sun) was consulted.

### Statistical analysis

Review Manager 5.3 software (available from the Cochrane Community, https://community.cochrane.org/help/tools-and-software/revman-5) was performed for data analysis [45]. Each study was evaluated by the Hardy–Weinberg equilibrium (H–W equilibrium, HWE) with a chi-square test (χ^2^ test) by IBM SPSS statistics 25 software [46]. Odds ratios (ORs) and their corresponding 95% confidence intervals (CIs) were utilized to test the association between each SNPs, rs2119882 and rs10830963 of MTNR1A/B and PCOS with six genetic models used to test the influence of genotype, that is, for rs10830963 (C> G): allelic (G vs. C), codominant (homozygote (GG vs. CC) and heterozygote (GC vs. CC) models), dominant (GG + GC vs. CC), recessive (GG vs. GC + CC) and superdominant (GG + CC vs. GC) models were calculated; and for rs2119882 (T> C): allelic (C vs. T), codominant (homozygote (CC vs. TT) and heterozygote (CT vs. TT) models), dominant (CC + CT vs. TT), recessive (CC vs. CT + TT) and superdominant (CC + TT vs. CT) models were calculated [47,48]. Our study used Cochrane Q-test for heterogeneity testing and the *I^2^*, a test statistic, for quantification [49]. In the Q-test, when *P*≥0.1 or *I^2^* ≤ 50%, the fixed-effects model could be established to analyze. Otherwise, the random-effects model could be used. Next, a Z-test was performed to evaluate the statistical significance of combined ORs in quantitative synthesis. And all analysis will use 0.05 as significantly statistical level. Finally, by eliminating the heterogeneous merger studies one by one, the effect of a single heterogeneous study on the entire model was further discussed through a sensitivity analysis. And Stata MP 16.0 software was used to analyze the publication bias by Egger’s and Begg’s tests.

## Results

### Study selection

**Figure 1:**
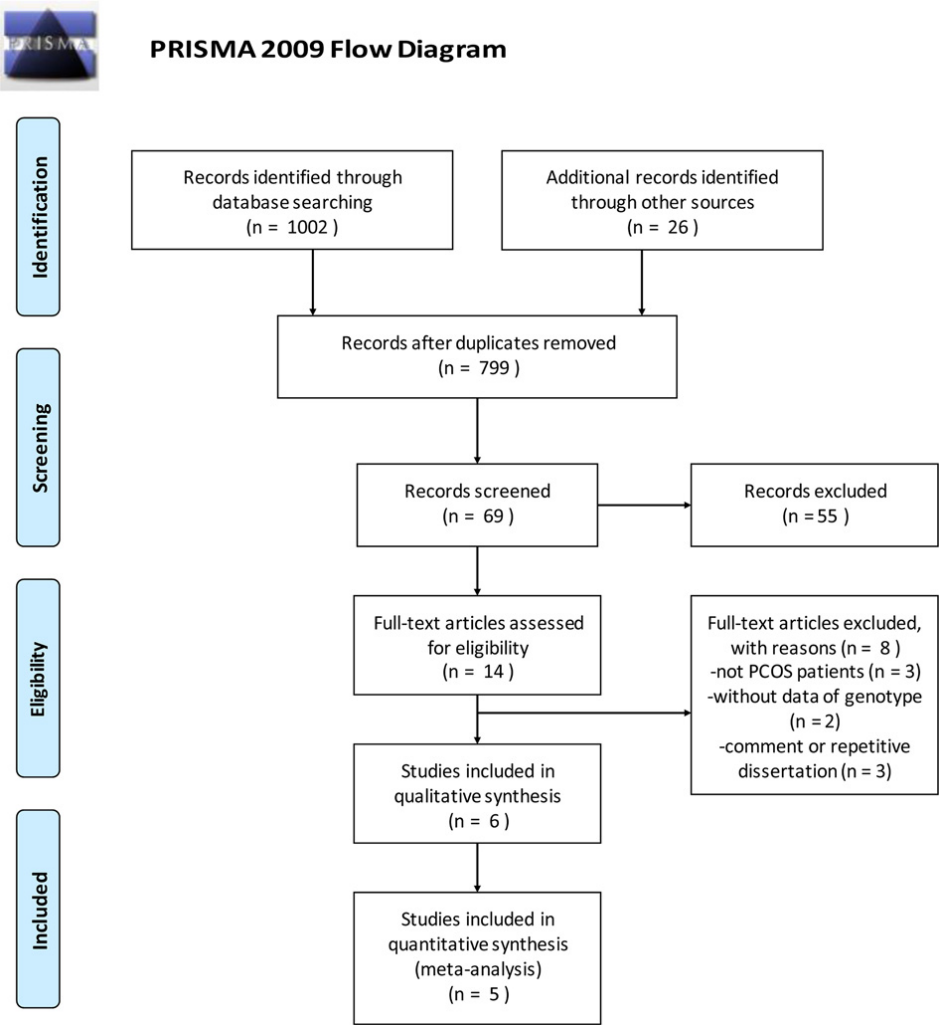
Flow diagram of selection process on genetic studies of MTNR polymorphisms with PCOS

In total, 1028 references were identified after searching from databases (refer to Supplementary Material S1, Search strategies) and their recommended links. Then, all searched reference lists were imported into EndNote X9, which helps to remove duplicate studies. The majority of articles were excluded for unrelated titles and abstracts and other eight articles were excluded because of which studied populations were not PCOS patients, genotype data were not valid, or references were unrelated. Additionally, Song et al. (2015) study [50] was removed because it was a family-based transmission disequilibrium test without control group. Finally, five articles met the quality requirements of meta-analysis and the flow diagram of our study selection is shown in Figure 1.

### Study characteristics

The eligible references in the present study involved two genes and six polymorphisms, MTNR1B gene (rs10830963, rs10830962, rs4753426, rs1562444, rs12792653) and MTNR1A (rs2119882). Four references, including Xu et al. (2019) [51], Yang et al. (2016) [52], Li et al. (2010) [53] and Wang et al. (2010) [39], reported the associations between rs10830963 and PCOS, including 1145 patients and 1408 controls. And two references, Xu et al. (2019) [51] and Li et al. (2011) [54], reported rs2119882, including 1680 patients and 1472 controls. Other SNPs had not been included in subsequent analysis due to insufficient data. All included studies with an NOS score more than 7, indicated a good level of quality (Table 1). The majority populations of the eligible references were Chinese and the methods used to detect gene polymorphisms were polymerase chain reaction (PCR) and sequencing. The studies characteristics are described in Table 2. Allele frequencies and genotypes of each study, and the outcome of HWE was listed in Table 3.

**Table 1:** The NOS

Study	Selection				Comparability	Exposure			Total Score
	Definition adequate	Representativeness	Selection of controls	Definition of controls		Ascertainment	Same method	Non-response rate	
Xu et al. (2019)	★	★		★	★★	★	★	★	8
Yang et al. (2016)	★	★		★	★★	★	★	★	8
Song et al.	★	★			★★	★	★	★	7
Li et al. (2011)	★	★	★	★	★	★	★	★	8
Li et al. (2010)	★	★	★	★	★	★	★	★	8
Wang et al. (2010)	★		★	★	★★	★	★	★	8

**Table 2:** Characteristics of the eligible studies in the meta-analysis

Study	Year	Ethnicity	Patients	MTNR	Study period	Stydy type	NOS	Case/control	Genotyping methods
Xu et al.	01/2019	Chinese	PCOS	MTNR1A, MTNR1B	March 2013–May 2015	Case–control	8	191+168/215	PCR- sequencing
Yang et al.	03/2016	Chinese	PCOS	MTNR1B	January 2012–May 2013	Case–control	8	182/196	PCR- sequencing
Song et al.	09/2015	Chinese, Han, Shandong	PCOS	MTNR1A, MTNR1B	July 2007–April 2014	Family trios	7	263(789)	PCR- sequencing
Li et al.	04/2011	Chinese, Han, Shandong	PCOS	MTNR1A	September 2006–February 2008	Case–control	8	481/522	PCR Tm-shift
Li et al.	10/2010	Chinese, Han, Shandong	PCOS	MTNR1B	February 2005–January 2007	Case–control	8	526/547	PCR Tm-shift
Wang et al.	11/2010	Chinese	PCOS	MTNR1B	/	Case–control	8	364/687	TaqMan-PCR

**Table 3:** Genotype and allele frequency of rs10830963 and rs2119882 in PCOS patients and controls

rs10830963	Group	Genotype (*n*)			χ^2^	*P*-value	Allele (*n*)		χ^2^	*P*-value
		CC	CG	GG			C	G		
Xu et al. (2019)	PCOS	125	169	65	30.002	0.000	419	299	29.351	0.000
	Control	114	91	10			319	111		
Yang et al. (2016)	PCOS	50	89	43	6.426	0.040	189	175	5.934	0.015
	Control	70	98	28			238	154		
Li et al. (2010)	PCOS	143	258	125	15.352	0.000	544	508	13.207	0.000
	Control	185	281	81			651	443		
Wang et al. (2010)	PCOS	126	185	53	1.201	0.549	437	291	0.746	0.388
	Control	229	340	118			798	576		

**rs2119882**	Group	Genotype (*n*)			χ^2^	*P*-value	Allele (*n*)		χ^2^	*P*-value
		TT	TC	CC			T	C		
Xu et al. (2019)	PCOS	68	165	126	16.446	0.000	301	417	17.507	0.000
	Control	69	97	49			235	195		
Li et al. (2011)	PCOS	167	215	99	9.962	0.007	549	413	9.824	0.002
	Control	215	236	71			666	376		

### Quantitative synthesis

**Figure 2:**
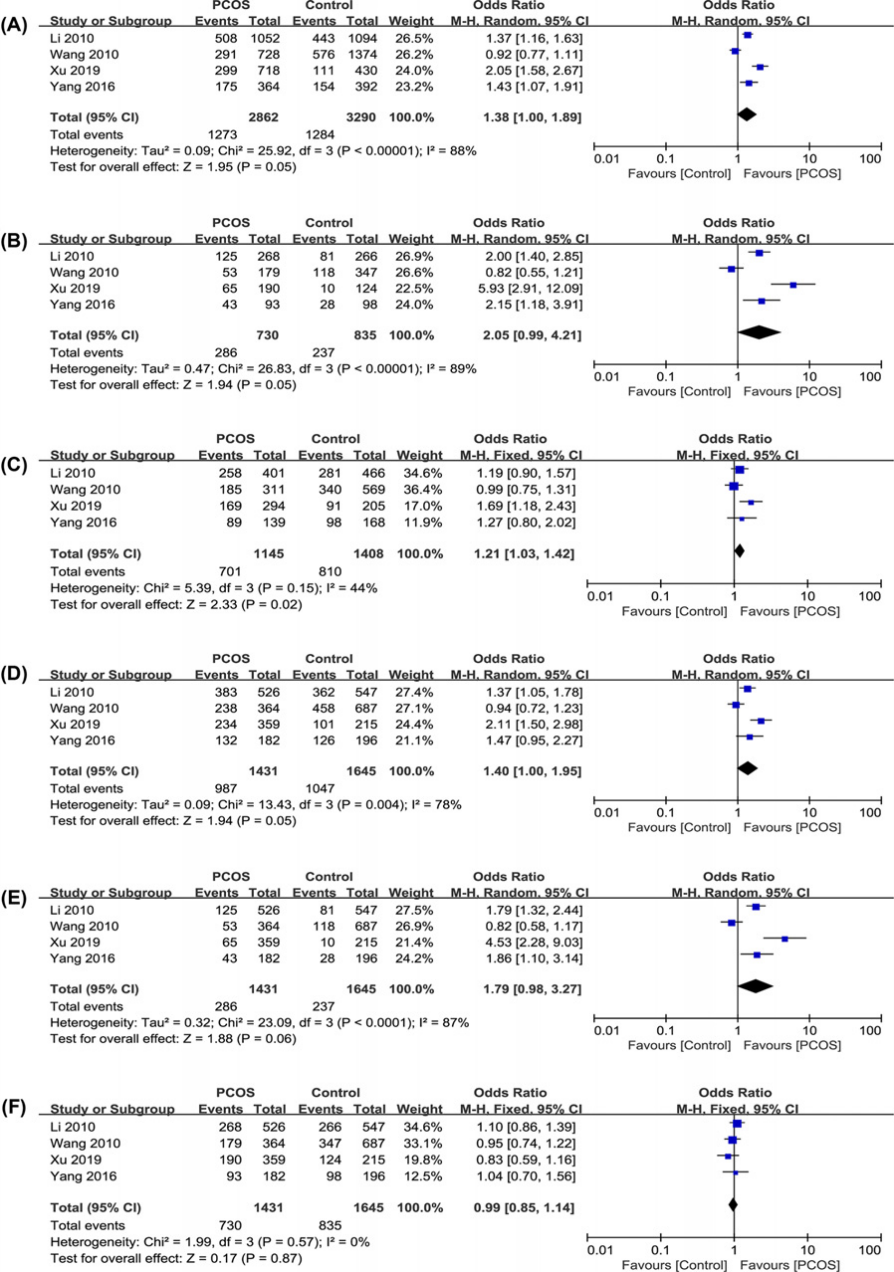
Forest plots of PCOS risk and the polymorphism of rs10830963 C>G in MTNR1B (**A**) G vs. C in allele. (**B**) GG vs. CC in homozygote. (**C**) GC vs. CC in heterozygote. (**D**) GG+GC vs. CC in dominant. (**E**) GG vs. GC+CC in recessive. (**F**) GG+CC vs. GC in superdominant.

**Figure 3:**
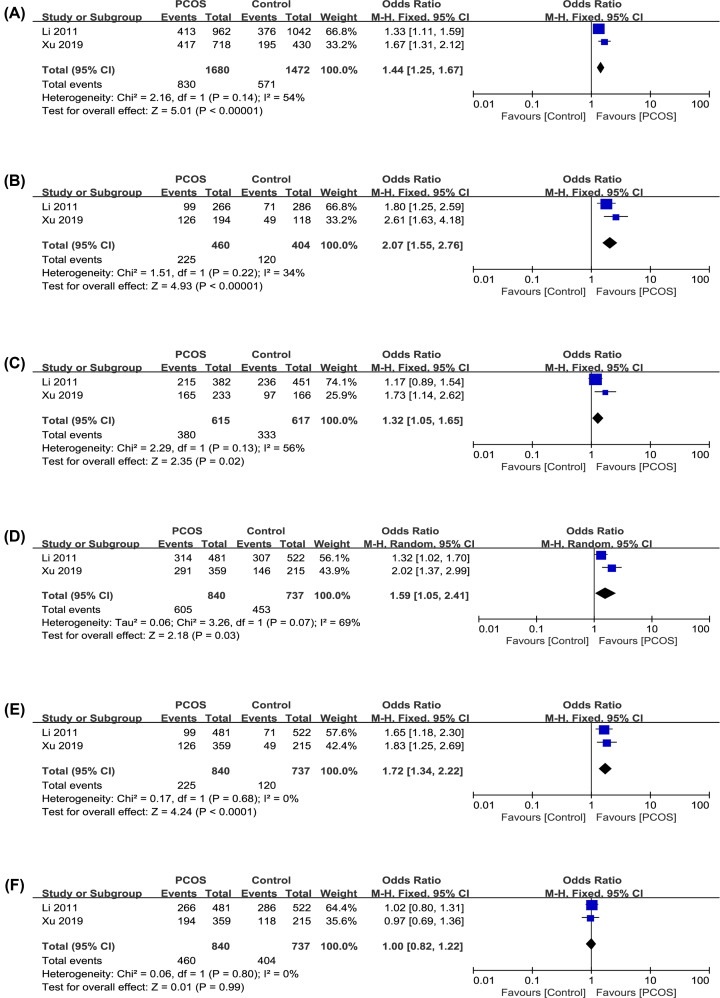
Forest plots of PCOS risk and the polymorphism of rs2119882 T>C in MTNR1A (**A**) C vs. T in allele. (**B**) CC vs. TT in homozygote. (**C**) CT vs. TT in heterozygote. (**D**) CC+CT vs. TT in dominant. (**E**) CC vs. CT+TT in recessive. (**F**) CC+TT vs. CT in superdominant.

The results of the association between the MTNR polymorphisms and the PCOS risk are shown in Table 4. The forest plots of each genetic model are described in Figures 2 and 3.

**Table 4:** Six genetic models of MTNR genes

		Allele				Homozygote				Heterozygote			
SNP	*n*	OR	95% CI	*P*	*I^2^*	OR	95% CI	*P*	*I^2^*	OR	95% CI	*P*	*I^2^*
rs10830963	4	1.38	[1.00, 1.89]	0.05	88%	2.05	[0.99, 4.21]	0.05	89%	1.21	[1.03, 1.42]	0.02	44%
rs2129882	2	1.44	[1.25, 1.67]	0.00	54%	2.07	[1.55, 2.76]	0.00	34%	1.32	[1.05, 1.65]	0.02	56%

		Dominant				Recessive				Superdominant			
SNP	*n*	OR	95% CI	*P*	*I^2^*	OR	95% CI	*P*	*I^2^*	OR	95% CI	*P*	*I^2^*
rs10830963	4	1.40	[1.00, 1.95]	0.05	78%	1.79	[0.98, 3.27]	0.06	87%	0.99	[0.85, 1.14]	0.87	0%
rs2129882	2	1.59	[1.05, 2.41]	0.03	69%	1.72	[1.34, 2.22]	0.00	0%	1.00	[0.82, 1.22]	0.99	0%

#### Analysis of rs10830963 polymorphisms in MTNR1B

To study the relationship between rs10830963 (C>G) and PCOS risk, we included four case–control studies (Table 3) and analyzed them with six genetic models [55] (Table 4, Figure 2). Among them, in the heterozygote model, the variation was significantly associated with PCOS occurrence (OR: 1.21, 95% CI: 1.03–1.42, *P*=0.02, *I^2^* = 44%). In the allelic, homozygote and dominant model, the variations were weakly associated to PCOS risk (OR: 1.38, 95% CI: 1.00–1.89, *P*=0.05, *I^2^* = 88%; OR: 2.05, 95% CI: 0.99–4.21, *P*=0.05, *I^2^* = 89%; OR: 1.40, 95% CI: 1.00–1.95, *P*=0.05, *I^2^* = 78%). In the recessive model, this mutation had a weaker association (OR: 1.79, 95% CI: 0.98–3.27, *P*=0.06, *I^2^* = 87%). And in last model, the superdominant model, no statistically significant association were found (OR: 0.99, 95% CI: 0.85–1.14, *P*=0.87, *I^2^* = 0%). All studies were evenly distributed on both sides of the center line, and no obvious publication bias was found.

#### rs2119882 polymorphisms analysis in MTNR1A

To study the association between rs2119882 and PCOS risk, we included two case–control studies (Table 3) and used six genetic models for analysis (Table 4, Figure 3). In five genetic models, allelic, homozygote, heterozygote, dominant, recessive and superdominant, significant statistical associations were found (OR: 1.44, 95% CI: 1.25–1.67, *P*<0.001, *I^2^* = 54%; OR: 2.07, 95% CI: 1.55–2.76, *P*<0.001, *I^2^* = 34%; OR: 1.32, 95% CI: 1.05–1.65, *P*=0.02, *I^2^* = 56%; OR: 1.59, 95% CI: 1.05–2.41, *P*=0.03, *I^2^* = 69%; OR: 1.72, 95% CI: 1.34–2.22, *P*<0.001, *I^2^* = 0%). However, in the superdominant model, no significant statistical association was found (OR: 1.00, 95% CI: 0.82–1.22, *P*=0.99, *I^2^* = 0%). All models had low or moderate heterogeneity, so the fixed-effect models were used to analyze them.

### Sensitivity analysis

**Figure 4:**
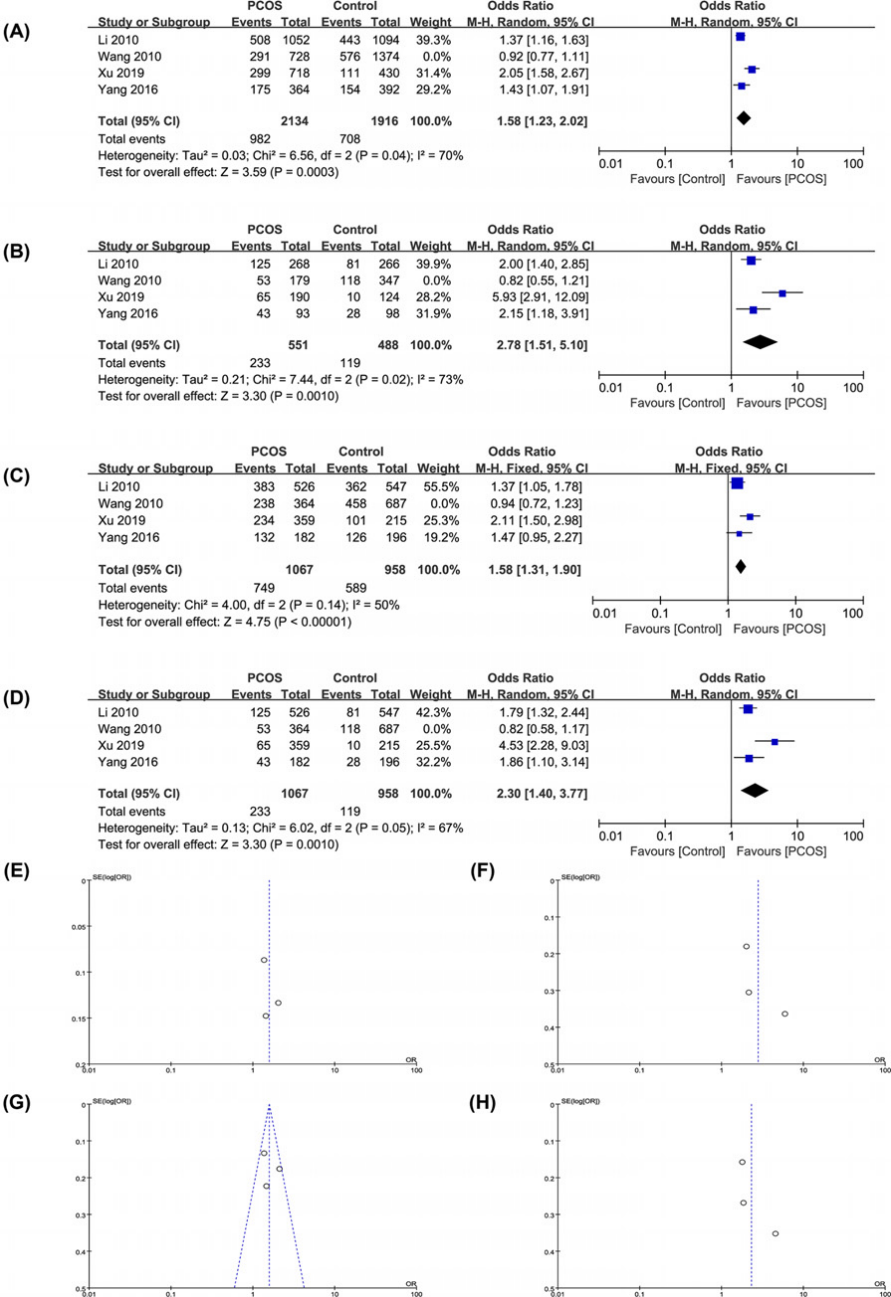
Forest plots and funnel plots of rs10830963 when Wang et al.’s study was removed (**A,E**) G vs. C in allele. (**B,F**) GG vs. CC in homozygote. (**C,G**) GG+GC vs. CC in dominant. (**D,H**) GG vs. GC+CC in recessive.

For genetic models with high heterogeneity (*I^2^* and *P*≤0.01), a sensitivity analysis was conducted by removing all the eligible references one by one in each model to assess the influence of each reference heterogeneity on the whole model analysis [56]. More details in forest plots and funnel plots are shown in Figure 4 and Table 5. After excluding the study of Wang et al. (2010) [39], the allelic, homozygote, dominant and recessive models in rs10830963 had significant statistical correlation with PCOS risk (OR: 1.58, 95% CI: 1.23–2.02, *P*=0.000, *I^2^* = 70%; OR: 2.78, 95% CI: 1.51–5.10, *P*=0.001, *I^2^* = 73%; OR: 1.58, 95% CI: 1.31–1.90, *P*=0.000, *I^2^* = 50%; OR: 2.30, 95% CI: 1.40–3.77, *P*=0.001, *I^2^* = 67%).

**Table 5:** Sensitivity analysis of rs10830963 C> G

Model	OR	95% CI	*P*	*I^2^*
Allele	1.58	[1.34, 2.02]	0.000	70%
Homozygote	2.78	[1.51, 5.10]	0.001	73%
Dominant	1.58	[1.31, 1.90]	0.000	50%
Recessive	2.30	[1.40, 3.77]	0.001	67%

### Publication bias

**Figure 5:**
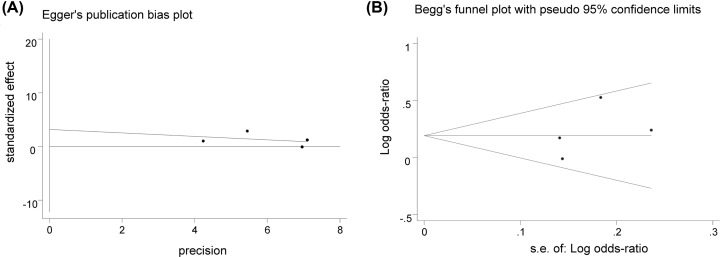
Egger’s test and Begg’s funnel plot of rs10830963 for heterozygote model (**A**) Egger’s regression test. (**B**) Begg’s funnel plot.

Due to the limited number of available references [44], publication bias was investigated only for the relationship between rs10830963 and PCOS risk by Stata MP 16.0 software. The result of heterozygote model (GC vs. CC) is shown in Figure 5. In Egger’s test, *P* was 0.469, while similarly in Begg’s test *P* was 0.497. Because both the *P*-values were more than 0.1, so no publication bias was found. Relationship between rs10830963 and PCOS risk was relatively stable and convincing.

## Discussion

As one among common endocrine disorders in gynecological reproduction, the PCOS pathogenesis is still unclear. Family studies revealed aggregation in PCOS [57], but no precise genetic mechanism can explain the etiology. Because these patients have hyperinsulinemia and IR, genes like MTNR1A/B related to insulin secretion possibly play a crucial role in PCOS progression. At the same time, recently several researches have been conducted or been registered [58] to determine the relationship between MTNR genes and PCOS susceptibility. But these researches have shown opposite results, so our study aims to further elaborate the relationship between MTNR1A/B gene and the risk of PCOS.

Melatonin works by activating two important G protein-coupled receptors, MT1 and MT2 [59]. By querying this National Center for Biotechnology Information (NCBI) database, it is known that the *MTNR1A* gene that encodes MT1 is located on the chromosome 4q35.2, and the *MTNR1B* gene of MT2 is located on chromosome 11q14.3. Rs10830963 is located in the intron region between the two exons encoding MTNR1B, while rs2119882 is located in the MTNR1A promoter region. Although these relative positions of SNPs in MTNR are different, the results for all genetic models in this two SNPs are still similar. However, the limitation of our study is that we only discussed from the perspective of MTNR gene while other SNPs are ignored. Because the pathogenesis of PCOS is complex and different genome-wide association studies (GWASs) conclusions are more heterogeneous [60], multiple SNPs mutations may affect metabolic and endocrine profiles in the same PCOS patient, such as insulin receptor gene (INSR) and luteinizing hormone/choriogonadotropin receptor (LHCGR) [61]. Therefore, further research is expected to combining multi-racial GWAS results of arrays by bioinformatics [62,63] and take other gene receptor into consideration to analysis-related molecular pathways [64].

In our meta-analysis, we find that in the heterozygote model, MTNR1B rs10830963 polymorphism is significantly associated with the PCOS occurrence. Through the sensitivity analysis, after excluding Wang et al. study [39], multiple model analyses showed that rs10830963 also has a significant correlation (the allelic, homozygote, dominant and recessive models). This may be due to the large heterogeneity of the present study. First, the study was conducted 10 years ago and its population was not in H–W equilibrium, possibly due to the bias in collecting participants in a limited geographical region. Second, the article does not report the period for recruiting the participants. Therefore, the study cannot rule out the effect of selection bias on the population. Finally, the SNPs are in a strong linkage disequilibrium and there may be a certain association between linkage markers. In addition, due to the large heterogeneity in different ethnicity, our study still needs large sample, multi-center and multi-ethnic studies to confirm.

Moreover, obese women in PCOS patients can be complicated with T2DM [65]. At the same time, their IR level is higher than normal, which can effectively predict impaired glucose tolerance and the occurrence of metabolic syndrome [66]. MTNR is also a candidate gene for IR and gestational diabetes mellitus [38]. So, further studies can take the clinical heterogeneity of PCOS into consideration and divide patients into different groups, such as obese and non-obese groups by body mass index [67], in following subgroup and phenotype analysis of SNPs.

As a result of weak endocrine regulation of melatonin through mutation receptor, to some extent, our meta-analysis supports the feasibility and acceptability of appropriate melatonin supplementation for PCOS [68,69]. At the same time, some studies have suggested that inositol can also be used to an novel and additional treatment of PCOS to improve symptoms [70–72], because as an insulin sensitizer it can decrease the levels of androgen, improve glucose metabolism [73], and restore spontaneous ovulation [74,75]. But due to the limited study of melatonin and inositol combination therapy [76,77], the clinical outcomes of IVF are still uncertain [78,79]. Therefore, the follow-up systematic review and meta-analysis is recommended to further confirm the combination therapy effect of melatonin and other drugs for PCOS.

## Conclusion

The SNPs rs10830963 and rs2119882 of MTNR1B and MTNR1A, are associated with a much higher PCOS risk in Chinese population. And above conclusions still require confirmation by future study including much larger multi-ethnic studies and other SNPs instead of the above conclusions.

## Data Availability

The analytical data are all included in the published articles and the final supplementary materials.

## Abbreviations

CI: confidence interval
H–W equilibrium/HWE: Hardy–Weinberg equilibrium
GWAS: genome-wide association study
IR: insulin resistance
IVF: *in vitro* fertilization
MTNR: melatonin receptor
NOS: Newcastle–Ottawa Scale
OR: odds ratio
PCOS: polycystic ovarian syndrome
SNP: single nucleotide polymorphism
T2DM: type 2 diabetes mellitus
